# Towards More Sustainable Schiff Base Carboxylate Anodes for Sodium-Ion Batteries

**DOI:** 10.3390/ma17194918

**Published:** 2024-10-08

**Authors:** Irene Gómez-Berenguer, Bernardo Herradón, José Manuel Amarilla, Elizabeth Castillo-Martínez

**Affiliations:** 1 Departamento de Química Inorgánica, Universidad Complutense de Madrid, 28040 Madrid, Spain; iregom12@ucm.es; 2 Instituto de Química Orgánica General, Consejo Superior de Investigaciones Científicas, 28006 Madrid, Spain; b.herradon@csic.es; 3 Instituto de Ciencia de Materiales de Madrid, Consejo Superior de Investigaciones Científicas, 28049 Madrid, Spain; amarilla@icmm.csic.es

**Keywords:** Schiff bases, anodes, sodium-ion batteries, sustainability, composite electrode processing

## Abstract

Bismine sodium salt (BSNa), a Schiff base with two sodium carboxylates, has shown promising electrochemical performance as an anode material. However, its synthesis involves toxic reagents and generates impurities, requiring significant solvent use for purification. This study introduces a novel synthetic method using sodium hydroxide as the sole reagent, which acts as both a base and Na source in the ion exchange step. With this procedure, we reduce the amounts of chemicals, diminish toxicity, improve the purity of the target compound, and use less solvent while maintaining comparable electrochemical performance. Additionally, the procedure is carried out under anhydrous conditions that avoid the undesirable hydrolysis of the imine linkages. In a previous report, the processing of the composite electrode was not established. In this article, we address this issue; the electrochemical performance, specifically the rate capability, is enhanced by processing the electrodes in laminate form rather than powder. As alternative to N-methyl-2-pyrrolidone (NMP), a common but disadvantageous solvent in laminate processing, other solvents were explored by testing acetone (DMK), methylisopropylketone (MIPK), and a DMK-NMP mixture. The remarkable electrochemical performance (specific capacity of 260–280 mAh/g, and capacity retentions higher than 84% at 1C (260 mA/g) remained consistent across these solvents. Furthermore, we investigated replacing copper with aluminum as the current collector to reduce costs and increase the energy density of the battery. While aluminum performed comparably to copper at low specific currents C/10 (26 mA/g), it showed a significant shift in the redox process potentials at higher specific currents.

## 1. Introduction

Lithium-ion batteries (LIBs) are the leading choice for portable consumer electronics due to their superior energy density. However, challenges arise when using LIBs in large-scale storage applications because of limited lithium reserves and its uneven geographic distribution. The cost of LIBs is heavily influenced by the scarcity of lithium (0.0017 wt% of the Earth’s crust), which exists only in combined form in nature due to its high reactivity. To address these issues, sodium, a low-cost, abundant (2.27 wt% of the Earth’s crust), and uniformly distributed element, is emerging as a potential replacement for lithium in sodium-ion batteries (SIBs) [1,2,3].

Another advantage of SIBs vs. LIBs is the possibility to use aluminum (Al) foil as the current collector for both the cathode and anode. In LIBs, the anode current collector must be made from copper (Cu) foil, which is more expensive and significantly heavier than Al [3]. This requirement is due to Al alloying with lithium at a low potential (close to 0 V vs. Li/Li^+^) [4], while it remains inert to sodium. Battery-grade aluminum costs three times less than battery-grade copper foil [5], although copper presents higher electronic conductivity [6]. In addition, the lighter weight of aluminum contributes to achieving a higher energy density in the battery.

Sodium ions have a larger radius compared to lithium ions (1.02 vs. 0.76 Å) [7], which causes greater volume changes during charge and discharge cycles and complicates the intercalation process [8,9]. This makes many anode materials that work well in LIBs less effective in SIBs [1].

Most SIB materials are inorganic, and their recycling is limited, requiring high energy and toxic chemicals, raising environmental concerns [10]. Organic electrode materials offer several advantages: They are composed of Earth’s abundant elements like carbon, hydrogen, oxygen, nitrogen, and sulfur, and their low atomic masses lead to high theoretical gravimetric capacities [11,12]. Furthermore, these materials can be sourced from biomass and processed at low temperatures, reducing environmental impact [10,11]. Organic materials also have flexible structures that accommodate larger sodium ions, allowing for efficient charge and discharge [13]. Their versatility and ease of modification enable the fine-tuning of their electrochemical properties [12]. Additionally, the redox mechanisms of organic materials can be adapted for multiple types of metal-ion batteries [10].

For organic anodes, three major functional groups (carboxylate, imine, and azo) are recognized for their suitability due to their low redox potentials [12]. Among the different types of organic electrode materials, carbonyl compounds containing C=O bonds have been the most extensively studied, owing to their unique multielectron reaction capabilities [13]. Despite the recent progress in organic electrode materials based on carbonyl compounds for energy storage, several challenges must be overcome before these materials can be commercialized. These include high solubility in electrolytes, which can result in capacity loss; low intrinsic electronic conductivity, which hampers performance; significant volume changes during cycling, which can compromise the structural integrity of the materials; and low tap density, which impacts the overall energy density of the batteries [11].

**Figure 1:**
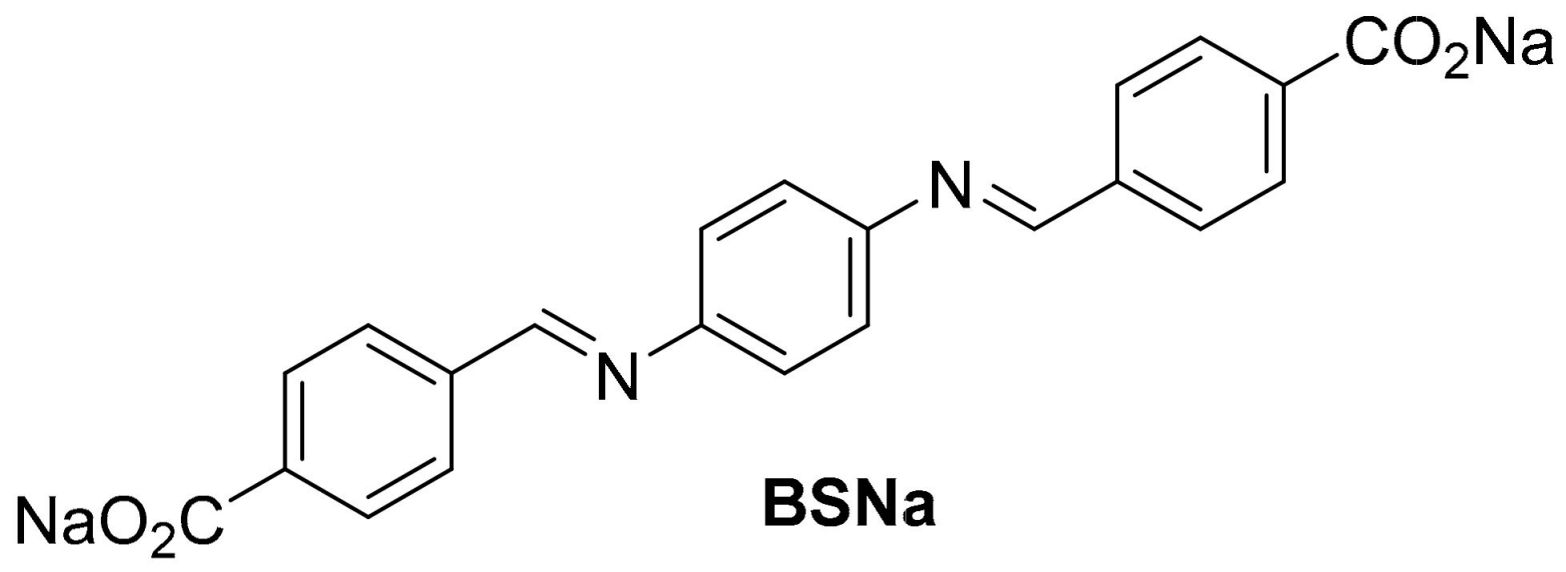
Structure of BSNa.

Alkali metal carboxylate networks, in particular, have been identified as efficient redox-active organic materials for ion insertion. The significant electronegativity difference within these compounds leads to highly ionic bonds, resulting in a broad array of structural possibilities. These materials combine the benefits of small organic units with the long-range ordering characteristic of networked structures [12,14]. The first example of carboxylates in SIBs was disodium terephthalate (Na_2_TP), whose effectiveness as an anode material for SIBs was simultaneously reported by two groups [15,16]. Additionally, Schiff bases, which feature imine groups, have also proven to be effective as anode materials due to their versatile redox behavior [17]. In 2015, López-Herraiz et al. [18] reported the electrochemical performance of a series of compounds containing both imine bonds and sodium carboxylates. The highest capacity and rate capability were achieved for the bisimine sodium salt (BSNa) shown in Figure 1. Additionally, BSNa demonstrated the advantage of being insoluble in most organic solvents, making it particularly suitable for use as an anode material.

The synthesis of BSNa is straightforward but it involves toxic reagents such as triethylamine, which also generate impurities. This indicates that there are aspects of the method that could be improved. In the original report, BSNa demonstrated reversible capacities close to the insertion of four Na ions per unit formula (258 mAh/g) at C/10, with a capacity retention of 97.5% after 25 cycles at C/10 and of 92% after 25 more cycles at C/5 within a potential window of 0.05–1.6 V. However, in that work, the electrochemical characterization was performed on powders comprising a blend of this compound and conductive carbons. Consequently, enhanced cycle life and rate capability are expected when evaluating these materials in the form of thin films prepared by casting (laminates) [18].

The electrode processing significantly influences battery performance, costs, and environmental impact [19,20]. The preparation of laminates involves several steps, including the mixing of the different components (active material, conductive additives, binder, and solvent) to create a slurry, casting it onto a current collector, and solvent evaporation [20,21]. Polyvinylidene fluoride (PVDF) is the most common binder, typically used with the solvent N-methyl-2-pyrrolidone (NMP) [22,23]. This solvent is facing increasing restrictions in several countries due to its toxicity and negative environmental impact [24,25]. Additionally, its relatively high boiling point (202 °C) and low autoignition temperature (252 °C) lead to elevated energy requirements and safety risks, respectively, during the electrode drying process. Alternative solvents such as dimethylsulfoxide (DMSO), dimethylformamide (DMF), cyrene, or γ-valerolactone (GVL) have been studied as replacements for NMP. However, these solvents present several challenges: DMSO can introduce sulfur impurities, cyrene and GVL result in poor adhesion, and DMF is highly toxic [26,27]. Additionally, these solvents have high boiling points; thus, the energy requirements are not eliminated. Aqueous processing is an alternative with many electrode materials, but imines undergo hydrolysis, and in principle, it cannot be processed with water. Other solvents, such as acetone (DMK) or methylisopropylketone (MIPK), which are less toxic than NMP and have the advantage of low boiling points, have not been tested for electrode processing, probably because their low boiling points imply other challenges during the drying process. A potential approach to the control evaporation rate is to use a mixture of solvents with a minimal amount of NMP. On the polymer side, the copolymers of PVDF and hexafluoropropylene (PVDF-HFP) have been reported to present better properties than PVDF, such as higher solubility and providing better mechanical strength to the final electrode [28].

The aim of the present work was to enhance the sustainability and performance of BSNa-based composite anodes for SIBs. This will be achieved by optimizing the synthesis process and improving electrode processing through the preparation of laminates, replacing NMP with acetone or MIPK, or decreasing its content. Additionally, the potential use of the aluminum current collector was also investigated.

## 2. Materials and Methods

### 2.1. Synthesis

The synthesis of bismine sodium salt (BSNa) involves a two-step process. In the first step, stoichiometric amounts of 4-formylbenzoic acid (2 equiv. mol, Aldrich) and *p*-phenylenediamine (1 equiv. mol, Aldrich) were mixed in absolute ethanol (LabKem) and subjected to magnetic stirring for 24 h. The resulting yellow powder (BSH) was then isolated by centrifugation at 8000 rpm, washed 2–4 times with absolute ethanol, and dried at 80 °C for 12–16 h. The second step was carried out using two different methods, both of which produced nearly quantitative yields:

- NaSCN method: BSH (1 equiv. mol) was reacted with triethylamine (Aldrich, 2 equiv. mol) in a vial with absolute ethanol. After a few minutes of stirring, NaSCN (Aldrich, 4 equiv. mol) was added to the suspension. The mixture was magnetically stirred for 24 h. The resulting yellow powder was centrifuged at 8000 rpm, washed six times with absolute ethanol, and dried at 60 °C for 12 h to yield BSNa-1. This yellow solid was further washed seven more times and dried at 60 °C for 12 more hours, yielding BSNa-2.
- NaOH method: BSH (1 equiv. mol) and NaOH (Scharlau, 3 equiv. mol) were stirred magnetically in a flask with absolute ethanol under reflux at 80 °C for 24 h. To remove the water formed during the reaction (as vapor in the azeotropic mixture with ethanol), molecular sieve (Alfa Aesar (Heysham, UK), 3 Å, 3–4 mm) was suspended over the reaction mixture in a Soxhlet-type device. The resultant yellow powder was centrifuged at 8000 rpm, washed four times with absolute ethanol, and dried at 80 °C for 12 h, yielding BSNa-3.

### 2.2. Characterization

Infrared (IR) spectra were measured in a Perkin Elmer UATR Two spectrophotometer (PerkinElmer, Shelton, CT, USA), with a 2 cm^−1^ spectra resolution and a total of 8 scans. Powder X-ray diffraction (PXRD) data were collected on a Bruker D8 Advanced diffractometer (Bruker, Billerica, MA, USA) with Cu Kα+K_β_ radiation (λ_α_ = 1.5405980 Å, λ_β_ = 1.5444260 Å) at 40 mV and 40 mA and a LynxEye SSD160-2 detector (Bruker). The patterns were registered in the range 2° ≤ 2θ ≤ 60°, with a step size of 0.016° and a total scan time of 1 h. For microanalysis, a LECO-932 analyzer (LECO Corporation, St. Joseph, MO, USA) with infrared cells and a conductivity detector was utilized to determine the elemental composition. Thermogravimetric analysis (TGA) was conducted using TA Q500 equipment (TA Instruments, New Castle, DE, USA); samples were heated at 10 °C/min from room temperature to 800 °C under nitrogen atmosphere. Scanning electron microscopy (SEM) was performed using a JEOL 6400 JSM (JEOL Ltd., Tokyo, Japan) or a JEOL JSM IT700HR microscope (JEOL Ltd.); the samples were coated with a thin film of gold prior to imaging. For transmission electron microscopy (TEM) the sample was placed on the grid without any solvent; imaging and compositional analysis by X-ray energy dispersive spectroscopy were performed in a JEOL JEM-2100 microscope (JEOL Ltd.) at an accelerating voltage of 200 kV, and the chemical mapping in STEM mode (scanning–transmission electron microscopy) was conducted using a JEOL JEM-3000F microscope (JEOL Ltd.) at an accelerating voltage of 300 kV.

### 2.3. Electrochemical Characterization

Composite electrode preparation involved generating a slurry composed of 70 wt% of active material (200–250 mg), 15 wt% of carbon black Super C-65 (Imerys, Paris, France), 5 wt% Ketjen Black (AkzoNobel, Amsterdam, The Netherlands), and 10 wt% poly(vinylidene fluoride-co-hexafluoropropylene) copolymer matrix (PVDF-HFP, trade name KF2801, Elf-Atochem, Toulouse, France). Prior to slurry formation, all components were dried in a vacuum furnace at 80 °C for 24 h. The slurry was then prepared using a solvent or a mixture of solvents, specifically 1-methyl-2-pyrrolidinone (NMP, Aldrich, St. Louis, MO, USA) as reference, DMK (Scharlau, Barcelona, Spain), methylisopropylketone (MIPK, Aldrich), and a mixture DMK-NMP (10% *v*/*v*). The resulting dispersion was cast using the doctor blade technique on a battery-grade Cu-foil or Al-foil current collector and dried at 70 °C for 2 h. Afterwards, the electrode was further dried overnight at 80 °C under dynamic vacuum. Disc electrodes of 1.13 cm^2^ were used as the working electrode with an active mass of 0.5–1.4 mg. The composite electrode was then assembled in two electrode CR2032 coin cells with a disk of metallic sodium as the anode and reference electrode. The Na disk was prepared by first mechanically cutting off the oxidized surface of a Na cylinder (Sigma Aldrich). The sodium was then placed inside a polypropylene bag, where it was carefully flattened using a roller. Finally, a 14 mm diameter disk was punched out using a die. The electrolyte 1M bis(trifluoromethylsulfonyl)imide (NaFSI, Solvionic, Toulouse, France) in 2-methyltetrahydrofuran (MeTHF, Aldrich) was prepared by dissolving the salt in the Me-THF within an aluminum bottle without stirring. A disk of Whatman BSF80 borosilicate glass fiber paper was used as separator, soaked with the electrolyte. Good interfacial contact was obtained by pressing all the coin cell components with a wavy spring between two stainless steel spacers. Half-cells were left to rest for 12 h at room temperature before starting the electrochemical measurements.

Cells were galvanostatically cycled using a Bio-Logic (BioLogic Spain, Madrid, Spain) or Arbin (Arbin Instruments, Houston, TX, USA) multichannel potentiostat/galvanostat in the potential range 0.05–1.6 V at 22 ± 2 °C. For the rate capability tests, the currents used were C/10, C/5, C/2 and 1C charge/discharge rates (1C = 258 mA/g). Five cycles were performed at each current intensity, followed by an additional five cycles at the initial intensity (C/10) at the end of the test. All voltages given in this paper are reported vs. the reference Na^+^/Na redox couple. For the rate capability analysis, the last cycle of each rate was selected (cycles 5, 10, 15, 20, and 25). In cases where cells exhibited anomalous behavior during these cycles, a more representative cycle was selected instead. Coulombic efficiency was calculated as the ratio of the charge capacity to the discharge capacity, expressed as a percentage.

## 3. Results and Discussion

### 3.1. Optimization of the Synthesis

#### 3.1.1. Synthesis and Characterization

**Scheme 1:**
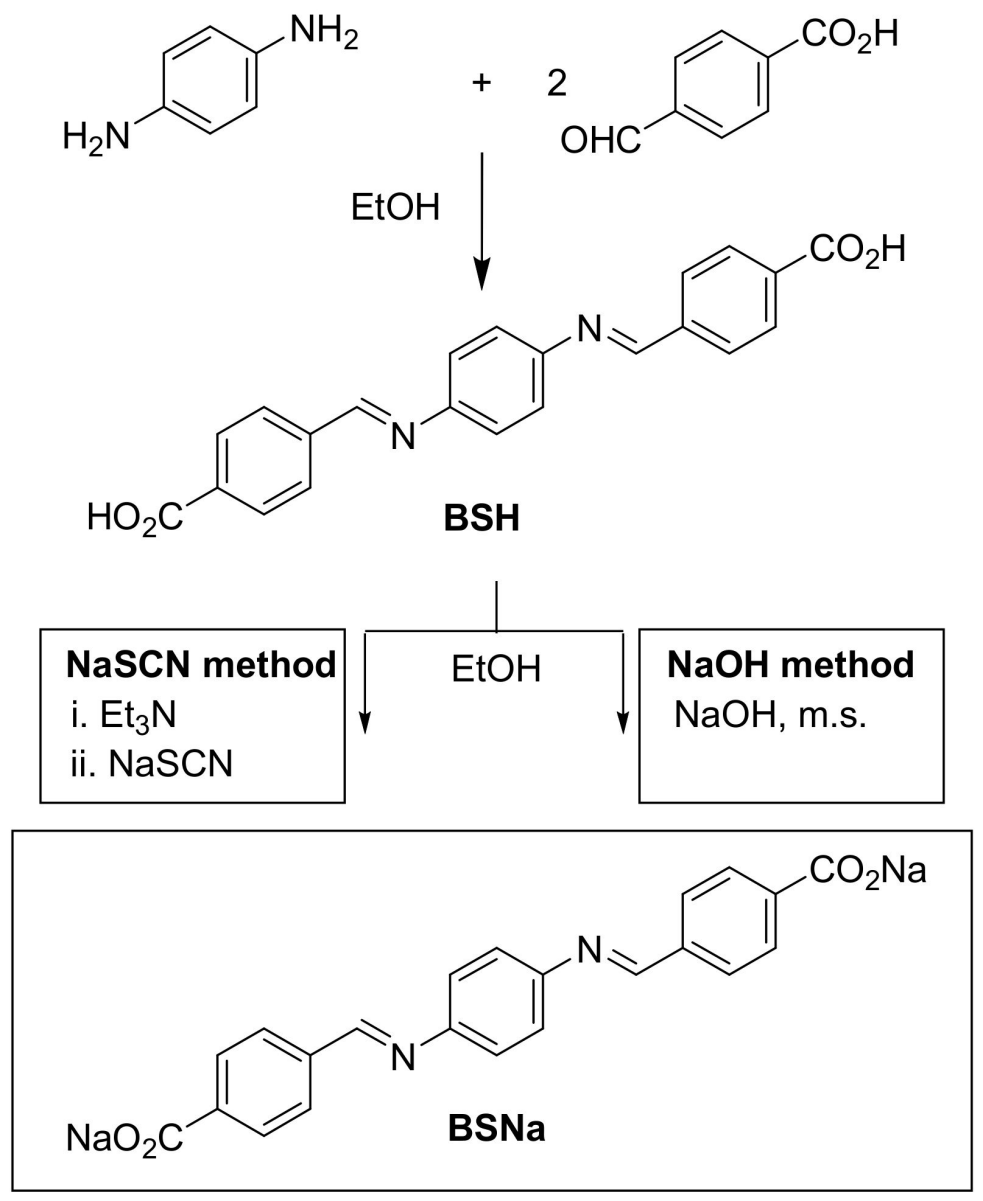
Synthesis of BSNa.

The synthesis of BSNa is shown in Scheme 1. First, a condensation reaction occurs between two equivalents of 4-formylbenzoic acid and one equivalent of *p*-phenylenediamine yielding the bisimine BSH. Subsequently, an ion exchange ensues, resulting in the formation of the sodium bisimine salt.

**Figure 2:**
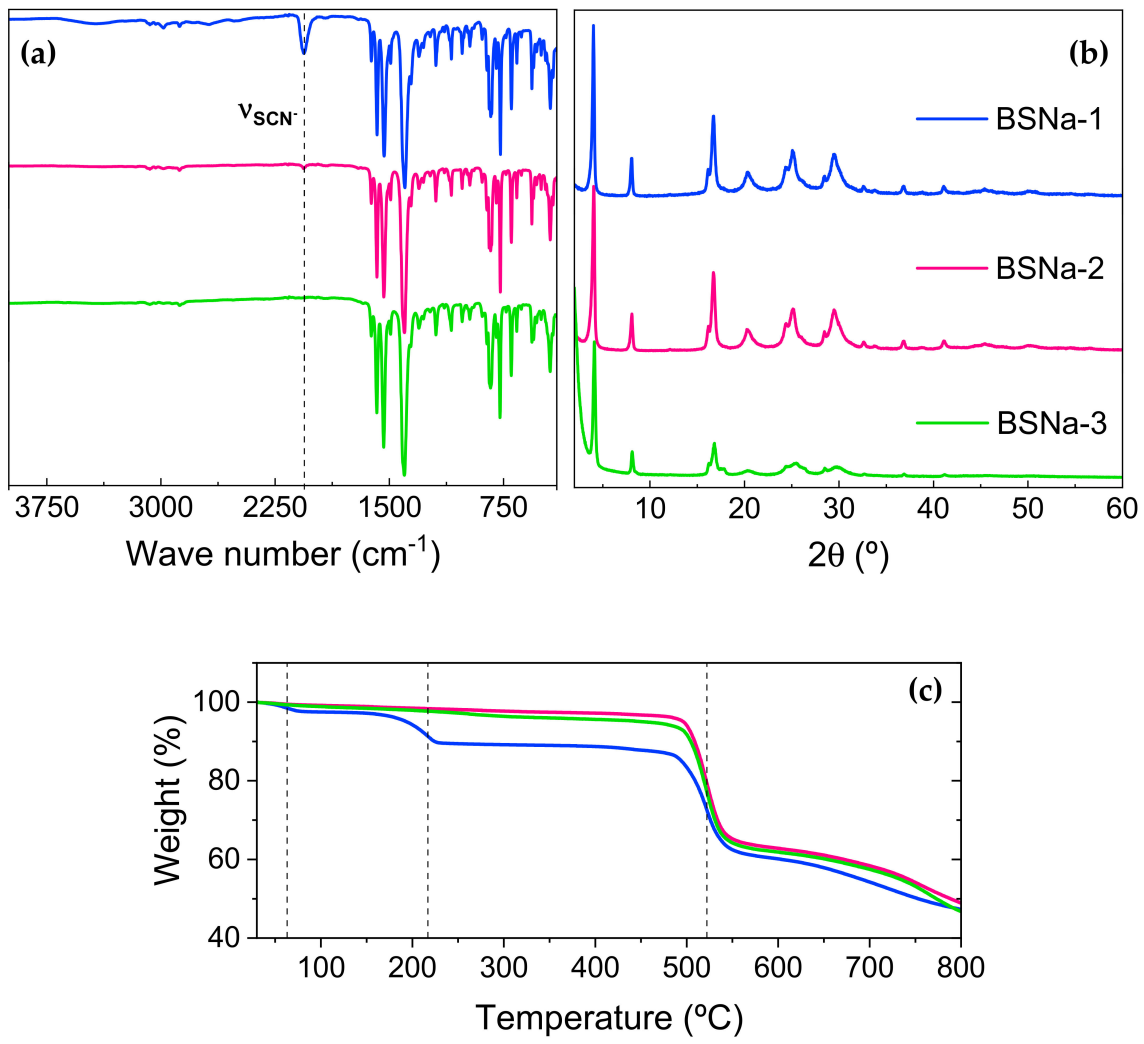
(**a**) Infrared spectra, (**b**) powder X-ray diffraction patterns, and (**c**) thermogravimetric curves of BSNa prepared using the NaSCN method after six washes (BSNa-1), after thirteen washes (BSNa-2), and using the NaOH method (BSNa-3).

In the original synthesis, toluene was used as the solvent in the first step, heating for 24 h hours at 85 °C [18]. In the second step, triethylamine was employed as a base to facilitate the deprotonation of carboxylic acids, with sodium thiocyanate serving as the cation donor and acetonitrile as solvent, heating for 24 h at 45 °C. In 2024, Castillo-Martínez et al. [29] synthesized the potassium analog salts (BSK), using ethanol as the solvent in both steps at ambient temperature, thus through a more sustainable route. When attempting to reproduce the BSNa synthesis in this work, using the method for BSK, it was observed that it was difficult to remove the thiocyanate anion. The work-up involved centrifuging the resulting solid and washing with ethanol. After six washes, a prominent peak at 2058 cm^−1^ in the IR spectra, corresponding to the SCN stretching, was observed (Figure 2a). After seven additional ethanol washes, the intensity of the SCN stretching decreased significantly but it was still detectable. This is consistent with the results from the microanalysis conducted to determine the content of carbon, nitrogen, hydrogen, and sulfur. The results indicate that, excluding the contribution of oxygen and sodium atoms, the sulfur content decreased from 4.25% after six washes down to 0.3% after thirteen washes. This could be due to the formation of the by-product triethylammonium thiocyanate as proposed by López-Herraiz et al. [18].

To save ethanol, avoid the formation of by-products, and reduce the quantity of toxic reagents (Et₃N and NaSCN), an alternative method using sodium hydroxide as the sole reagent was deployed. The ion exchange process with NaOH had already been employed for the synthesis of sodium terephthalate [16]. In our method, water is the only by-product, and it was captured from the vapor phase using a molecular sieve to prevent the degradation of the bisimine product, which hydrolyzes in water. As shown in Figure 2a, the infrared spectra indicate no significant differences between the sample prepared with the NaSCN method and the NaOH method, except for the absence of the SCN stretching band. There is no difference either in the positions of the reflections in the powder X-ray diffraction patterns of the samples produced via both methods (Figure 2b), indicating that the thiocyanate impurity does not affect the crystalline structure adopted by the hybrid sodium salt. However, there is a marked difference in the relative and absolute intensity of the reflections, with the material obtained using the NaOH method exhibiting significantly lower intensity, as the background signal from the direct beam is more clearly seen, meaning lower crystallinity. This aspect will be further discussed.

The presence of residual impurities via the NaSCN method is further confirmed through thermogravimetric analysis (TGA) (Figure 2c). The TGA of the BSNa-3 obtained via the NaOH method and the BSNa-2 from the NaSCN method after thirteen washes exhibited similar thermal stability, with a significant mass loss occurring only at 522 °C, which is assigned to the thermal decomposition of the product BSNa. In contrast, the BSNa obtained using the NaSCN method after only six washes displays additional mass losses at 217 °C and 63 °C, and none of them seems to be due to residual NaSCN, which shows its first significant mass loss at 767 °C, (Figure S1). This observation effectively rules out the possibility that the thiocyanate infrared signal and the sulfur content detected in the microanalysis originate from the starting reagent NaSCN. Instead, it confirms the formation of a by-product, likely triethylammonium thiocyanate.

**Figure 3:**
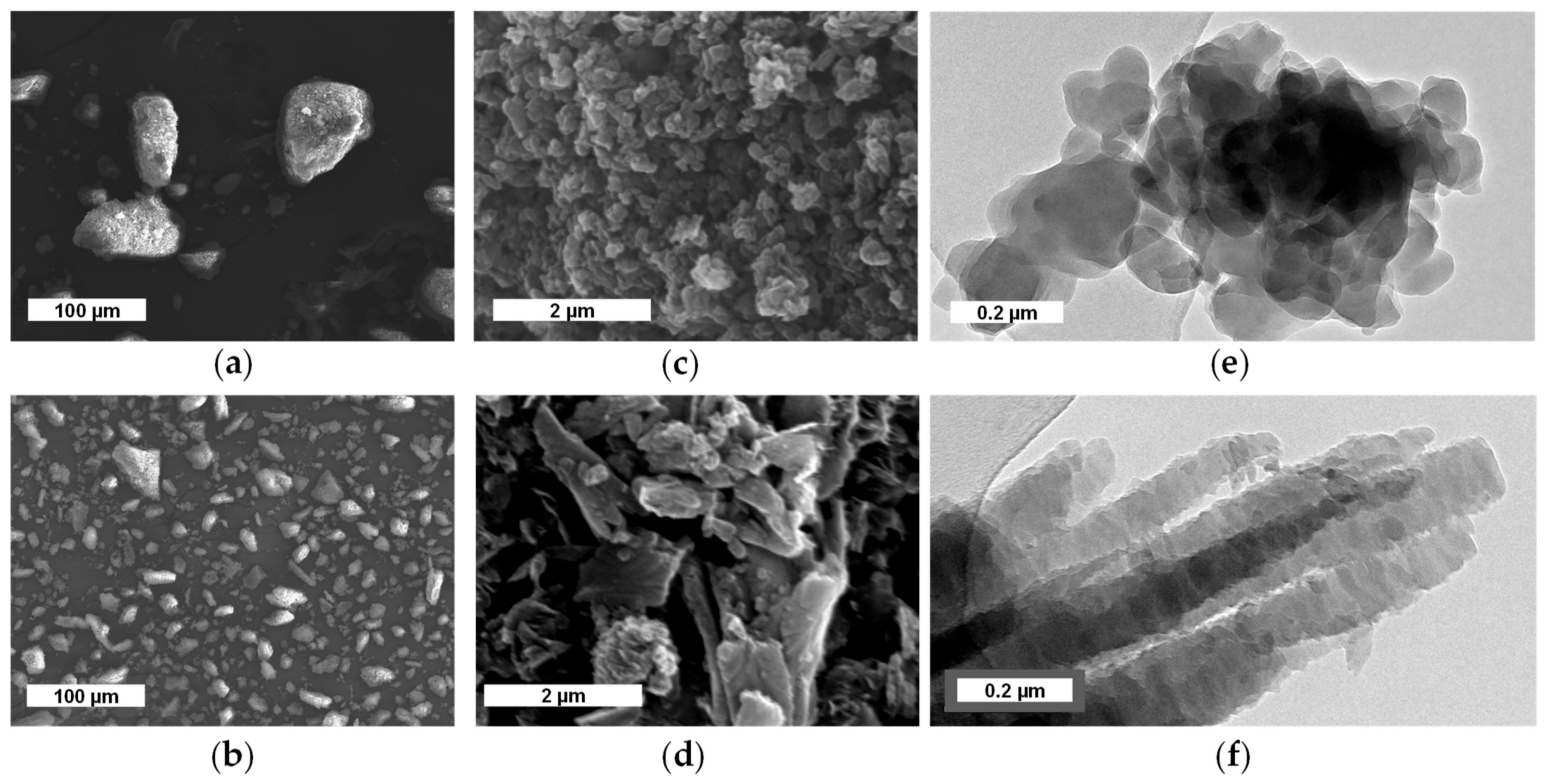
SEM and TEM images of BSNa 2 (**a**,**c**,**e**) and BSNa-3 (**b**,**d**,**f**).

SEM images reveal morphological differences between the materials produced by the two methods (Figure 3). The NaOH method results in smaller BSNa agglomerates compared to those produced using the NaSCN method, as shown in Figure 3a,b. At higher magnification, distinct differences in particle shape are observed; BSNa-2 (Figure 3c) exhibits popcorn-like particles, while BSNa-3 (Figure 3d) shows a scale-like morphology. High magnification TEM images further illustrate these distinctions; Figure 3f shows that the scale-like particles are composed of nanometric crystals nanostructured as filaments. In contrast, the TEM image in Figure 3e demonstrates that particles produced using the NaSCN method, though also nanometric, are slightly larger, more isotropic and do not exhibit a specific growth direction.

Crystallite size was determined from the width of the most intense diffraction peak (~4°) of the X-ray powder diffraction patterns for each material, in conjunction with the Debye–Scherrer equation [30]. This reflection was selected because it seems to be a single reflection with no overlap of several reflections, but the resulting size is of course only indicative of the crystal size along one specific crystallographic direction. For the BSNa-1 synthesized via the NaSCN method, the crystallite size measured 30 nm after six washes with ethanol (BSNa-1), increasing to 39 nm after thirteen washes. Although not a very large difference, this suggests that crystallite size may increase as the thiocyanate content decreases, which in turn suggests recrystallization to occur during ethanol washing despite the very low solubility of the salt in all tested solvents. Nonetheless, this difference seems to only occur in the first reflection, as all others match very well in intensity before and after washing. In comparison, the BSNa synthesized using the NaOH method (BSNa-3) presents a crystallite size of 42 nm as deduced from this first reflection, closely matching that of BSNa-2, despite all other reflections seeming less intense and broader. These findings align with the TEM observations (Figure 3e,f), which indicates that the primary particles in both BSNa-2 and BSNa-3 are nanometric, with sizes below 100 nm.

Figure S2 presents the SEM images of BSNa-1, which closely resemble those of BSNa-2 shown in Figure 3. This similarity suggests that variations in thiocyanate concentration (in both BSNa-1 and BSNa-2) do not significantly influence the morphology of the materials. Additionally, elemental mapping (Figure S3) indicates that all elements, including sulfur in BSNa-1, are uniformly distributed across the three materials and are not concentrated in large, distinguishable particles.

#### 3.1.2. Electrochemical Performance

**Figure 4:**
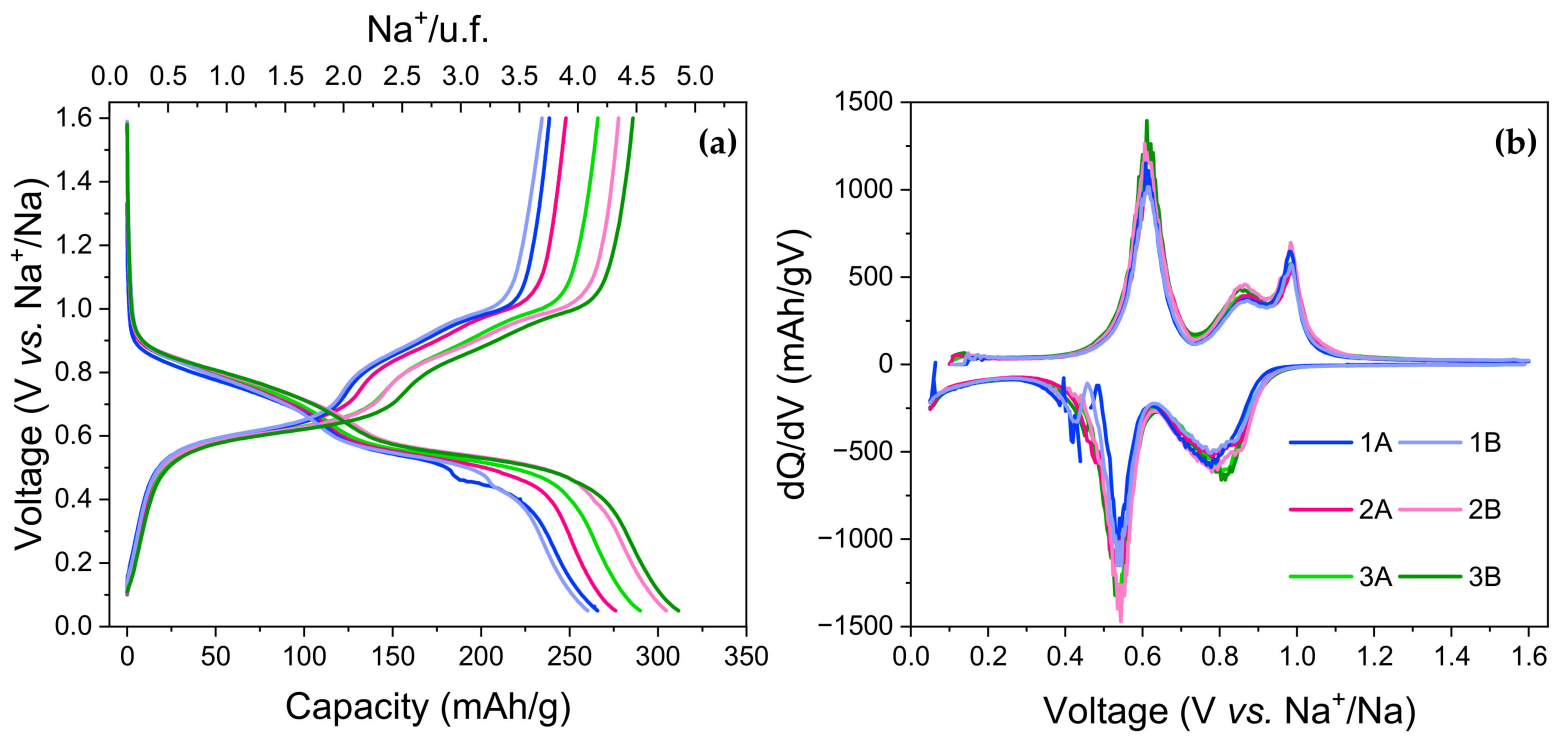
(**a**) Voltage vs. specific capacity and (**b**) voltage derivatives of the specific capacity vs. voltage for the second cycle at C/10 of BSNa-1 (1), BSNa-2 (2), and BSNa-3 (3). A and B are twin cells.

Galvanostatic experiments were conducted for the three synthesized BSNa-samples in duplicate within the voltage range of 0.05–1.6 V vs. Na^+^/Na. Figure S4 depicts the voltage vs. capacity during the first cycle at a C/10 rate (26 mA/g) for all three BSNa samples. The experimental results demonstrate a significant irreversible capacity during the first reduction that is not recovered during subsequent oxidation (about 63% of irreversible capacity loss for the three samples). This phenomenon aligns with previous findings [18], where the reported substantial irreversibility in the first cycle was attributed to electrolyte decomposition and the formation of a solid–electrolyte interface (SEI). The possible occurrence of secondary reactions was also suggested, potentially involving radical anions forming during the initial process. The coulombic efficiencies for the first cycle in that report were already notably low, ranging from 33 to 39%. Figure 4 shows the voltage profile for the second galvanostatic cycle, highlighting a significant enhancement in coulombic efficiency, which increases from an initial range of 33–39% in the first cycle to 90–92% in the second cycle. Figure 4a depicts three distinct redox processes. During oxidation, these processes occur at 0.62 V, 0.86 V, and 0.98 V vs. Na^+^/Na (Figure 4b). In the reduction, the first process appears at 0.53 V, while the latter two processes merge into a single broad band at 0.78–0.81 V. It is evident that the material with the highest amount of SCN impurity exhibits the poorest performance. Moreover, sometimes the cells showed an additional feature in the voltage profile at 0.4 V vs. Na^+^/Na. This is better seen in the dQ/dV (Figure 4b) and suggests that the low voltage redox process is split into two, probably due to some kinetic hindrance. It is important to note that no significant differences were observed between the material obtained using the NaSCN method after thirteen washes and the one produced via the NaOH method.

Table 1 lists the average specific capacities and their standard deviations for each material. The small mass of active material (0.5–1.1 mg) affected the accuracy of the weight measurements, leading to a deviation of ~5% between the duplicate cells.

**Figure 5:**
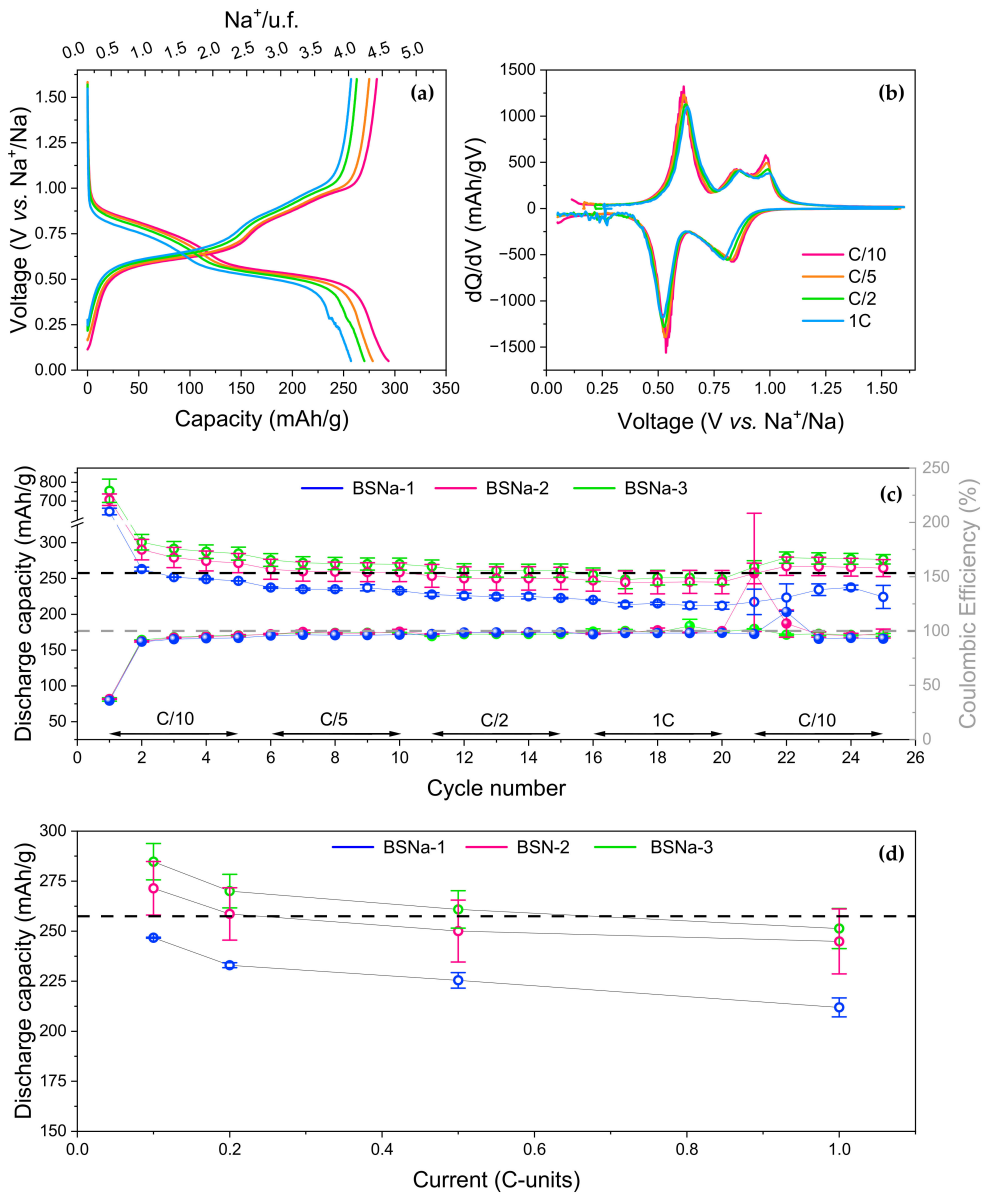
(**a**) Voltage vs. specific capacity and (**b**) voltage derivatives of the specific capacity vs. voltage for the best performing cell of BSNa-3 prepared using the NaOH method; (**c**) average of and deviation in specific discharge capacity (open circles) and coulombic efficiency (spheres) vs. cycle number of BSNa-1, BSNa-2, and BSNa-3. Theoretical specific capacity (258 mAh/g) and optimum coulombic efficiency (100%) are marked with dashed lines. (**d**) Average of and deviation in specific discharge capacity vs. current of BSNa-1, BSNa-2, and BSNa-3.

The results of the rate capability experiments are presented in Figure 5 and Table 2. Figure 5a,b represent cell BSNa-3B assembled with the NaOH-derived material, which yielded the best results. In Figure 5c, for BSNa-1, the coulombic efficiency values for cell B only are presented, as cell A exhibited anomalous behavior during the charge cycles. As observed in the first two cycles, BSNa-1 exhibits the lowest capacity values, while the other two materials display very similar performances with high capacity values, close to 280 mAh/g at C/10.

The average capacities of each material, along with their deviations, are recorded in Table 2. All three BSNa samples demonstrate high capacity retention at high current rates, retaining 85–90% of their nominal capacity when cycled from C/10 to 1C. This slight decrease in capacity is likely attributed to kinetic factors. Notably, when the current is returned to C/10 in the last five cycles, the capacity is recovered in all cases (Figure 5d).

From these results, it can be concluded that although it is possible to produce BSNa with ethanol (instead of toluene) at room temperature, the presence of the thiocyanate impurity adversely affects the electrochemical performance. The similar electrochemical performance of BSNa-2 and BSNa-3 indicates that the morphological differences observed in Figure 3 do not significantly influence their electrochemical behavior. The NaOH method is identified as the superior synthesis approach as it conserves ethanol, avoids by-product formation, and reduces the quantity and toxicity of reagents.

### 3.2. Electrode Processing

#### 3.2.1. Solvents

In order to replace the NMP and enhance the sustainability and cost-effectiveness of electrode processing, DMK and MIPK have been studied as alternative solvents. These solvents are less toxic and possess lower boiling points (56 °C and 94 °C, respectively) compared to NMP (202 °C). Additionally, a mixture of DMK and a small amount of NMP (10% *v*/*v*) was also tested. According to the results above, the active material employed for these electrodes was produced using the NaOH method (BSNa-3).

Four laminates were prepared (Figure S5) using a blade height of 250 µm. When using DMK as the solvent, its low boiling point necessitates very rapid processing in general, which is difficult to achieve in practical applications. This requirement adds complexity to the manufacturing process. In contrast, when using MIPK, the higher boiling point mitigates evaporation issues; however, the slurry’s adhesion to the current collector is poorer. This poor adhesion presents a challenge in utilizing the majority of the laminate, as cutting disks (which are used as electrodes in the cell) can cause the edges of the laminate to detach from the current collector. In the case of the DMK-NMP mixture, the addition of a small amount of NMP (10% *v*/*v*) effectively prevents the rapid evaporation problem associated with DMK alone and ensures good adhesion to the current collector. This results in a well-formed and manageable laminate, combining the benefits of the DMK’s lower toxicity and the reduced volatility and improved adhesion provided by the presence of NMP.

However, a disadvantage shared by all four laminates, including the one with NMP, is the small mass of active material obtained from the disks (0.5–1.4 mg). The larger masses correspond to the NMP laminate, while the lower masses are associated with the MIPK and DMK laminates. This small mass results in significant deviations in measurements due to the weighing errors, impacting the accuracy and reliability of the results. Increasing the blade height could potentially increase the mass, but this might also cause the laminates to fragment more due to the increased thickness, thereby complicating their handling and processing.

Figure S6 shows the infrared spectra and powder X-ray diffraction patterns of the composite electrode powders obtained by mixing the different electrode components and evaporating the solvent. The IR spectra are consistent across electrodes prepared with different solvents, matching the spectrum of the BSNa powder. The PXRD patterns reveal that the characteristic peaks of the BSNa are present in the composite electrodes, indicating that the active material remains unmodified during the processing, as also suggested by the IR spectra. However, a reduction in peak intensity, particularly in the first reflection of the PXRD patterns for all cast composite electrodes, indicates a loss of crystallinity compared to pure BSNa-3. This reduction in crystallinity can be attributed to the incorporation of other electrode components.

**Figure 6:**
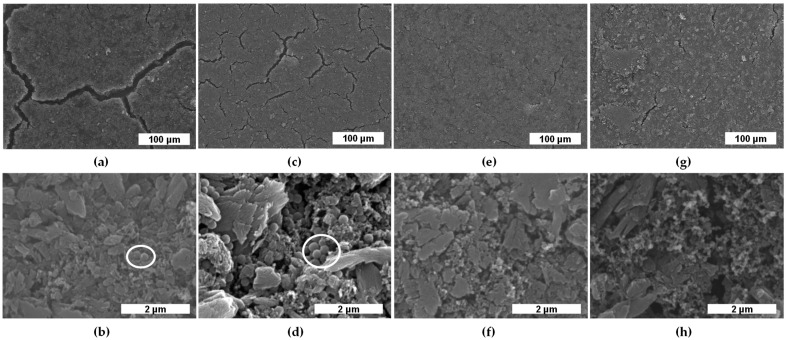
SEM images of electrodes prepared with (**a**,**b**) DMK, (**c**,**d**) MIPK, (**e**,**f**) DMK + NMP, and (**g**,**h**) NMP. White circles in (**b**,**d**) highlight spherical PVDF-HFP particles.

The SEM images of cast electrodes (Figure 6) reveal differences among the composite electrodes. When acetone was used as the solvent, the resulting electrode exhibited wide cracks (Figure 6a). In contrast, the electrode prepared with MIPK showed narrower and shorter, but more numerous cracks (Figure 6c). The electrodes produced with NMP and the DMK-NMP mixture displayed fewer cracks overall (Figure 6e,g). These variations are likely related to the evaporation rate of the solvents. Solvents with lower boiling point evaporate more quickly, leading to increased cracking during the electrode formation process. Interestingly, when the magnification is increased, the scale morphology of the BSNa-3 particles is maintained after processing regardless of the solvent used (Figure 6b,d,f,h).

**Figure 7:**
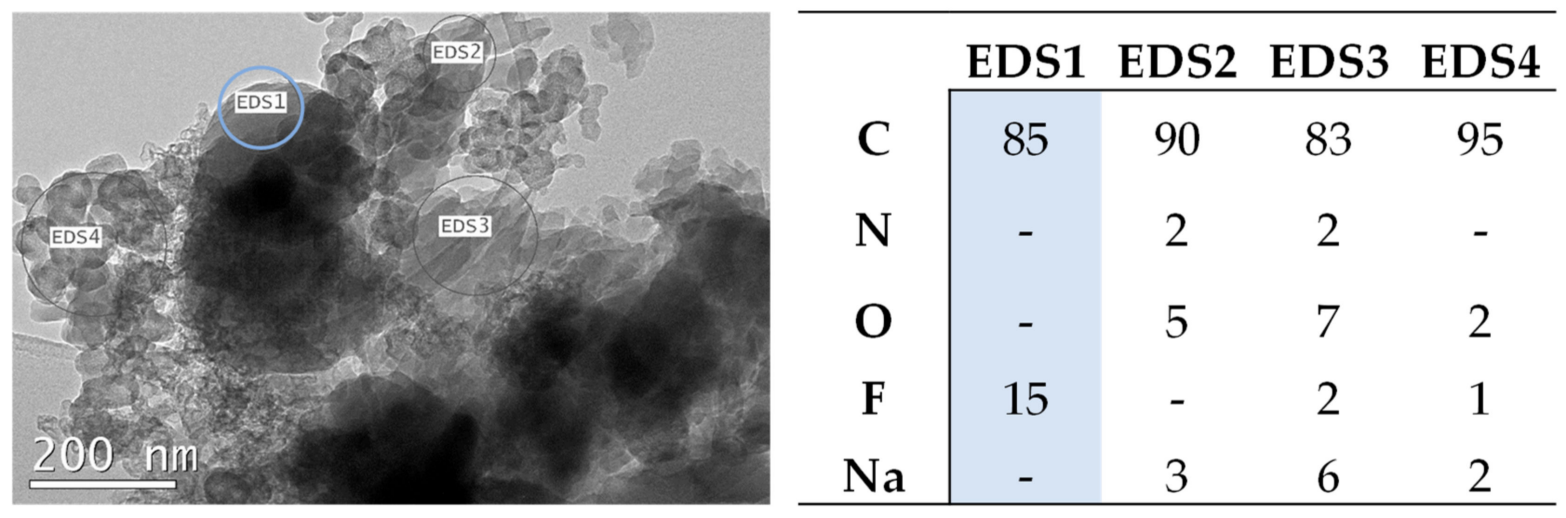
EDS-TEM of the MIPK laminate. EDS1 blue circle correspond to the spherical particles of PVDF-HFP copolymer as determined by EDS analysis.

Another clear difference among laminates is the presence of spherical particles in the DMK and MIPK electrodes (Figure 6b,d). These particles are absent in the electrodes prepared with NMP and the DMK-NMP mixture (Figure 6f,h). EDX analysis (Figure 7) and elemental mapping (Figure S7) in the TEM reveal that these spherical particles (EDS 1 in Figure 7) are composed solely of fluorine and carbon, which suggests that the spherical particles are the PVDF-HFP copolymer, which also has spherical morphology (Figure S8). The polymeric binder distribution is, thus, uneven in the DMK and MIPK laminates. In contrast, it appears uniform in the DMK-NMP and NMP laminates, suggesting that NMP promotes a more homogenous polymer distribution throughout the laminate. This uneven distribution may be linked to the increased cracking and poor adherence observed in the DMK and MIPK laminates. Since PVDF-HFP is highly soluble in both DMK and MIPK, more so than in NMP, the uneven polymer distribution is not likely due to solubility issues, and its presence is quite intriguing.

The electrochemical performance of the laminates was measured against sodium metal in duplicate. Figure S9 shows the first reduction for each laminate. As seen in Figure S9b, the redox processes occurring during the first reduction are almost identical, indicating that the choice of processing solvent does not influence the redox mechanism of the first reduction.

All four laminates exhibit low coulombic efficiencies in the first cycle, ranging from 32 to 39% (Table 3), which are comparable to the values reported in powdered form [18]. This is consistent with the well-established issue of first-cycle irreversible loss, a significant challenge for anode materials in sodium-ion battery systems [31,32] that is still not solved in these Schiff bases with current synthetic and processing approaches. Recently, pre-sodiation strategies, including chemical and electrochemical methods, are being considered for effective ways to increase the coulombic efficiency of the anode during the first cycle [33].

**Figure 8:**
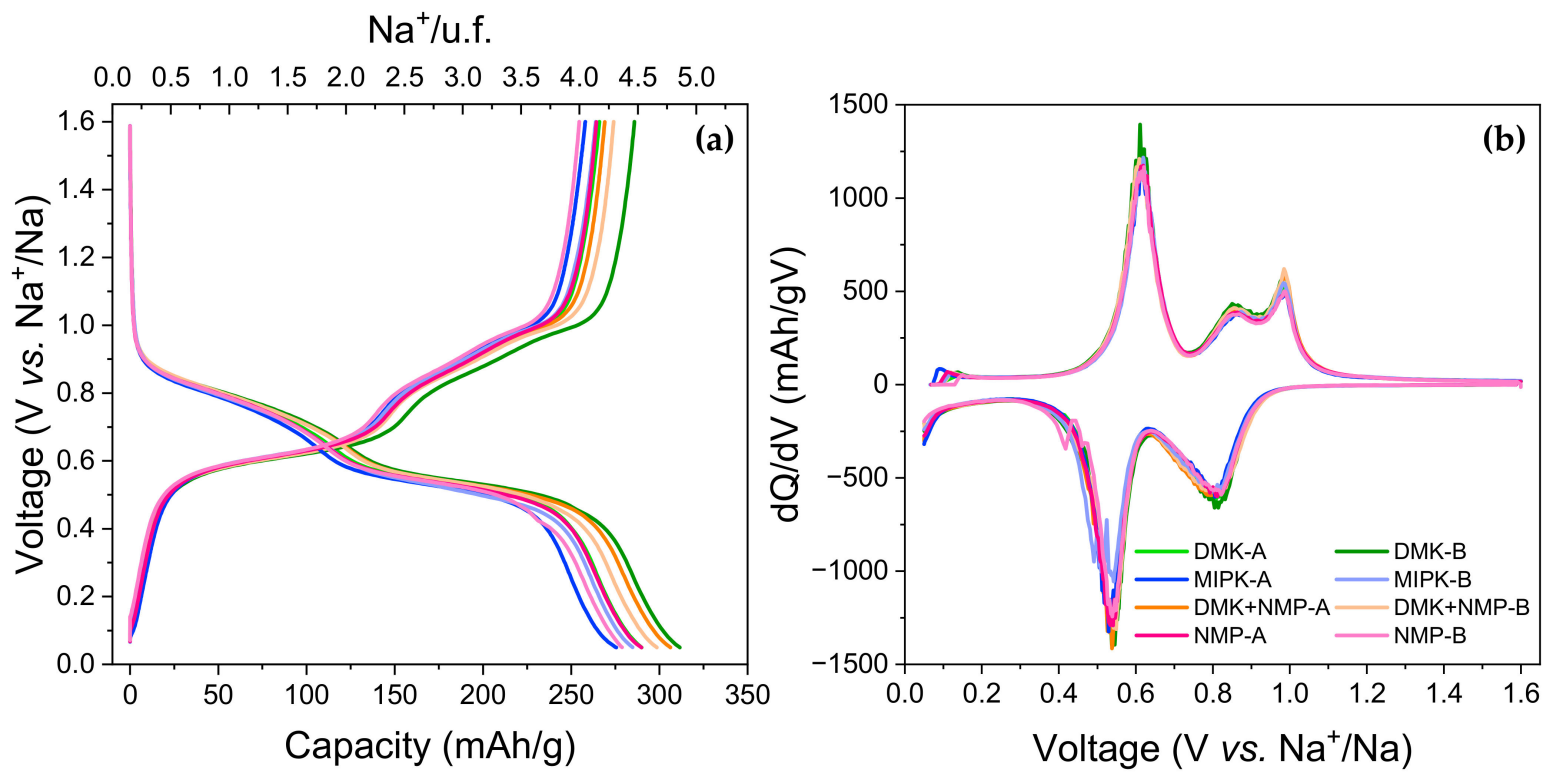
(**a**) Voltage vs. specific capacities and (**b**) voltage derivatives of the specific capacity vs. voltage for the second cycle (C/10) of the four laminates.

The charge/discharge curves of the second cycle, illustrated in Figure 8, suggest that the MIPK and NMP laminates yield slightly lower reversible capacities along with low voltage peak splitting upon reduction. No appreciable differences are observed between electrodes prepared with DMK and DMK-NMP. Figure 8b also shows that the dQ/dV profile is practically identical for the four laminates.

**Figure 9:**
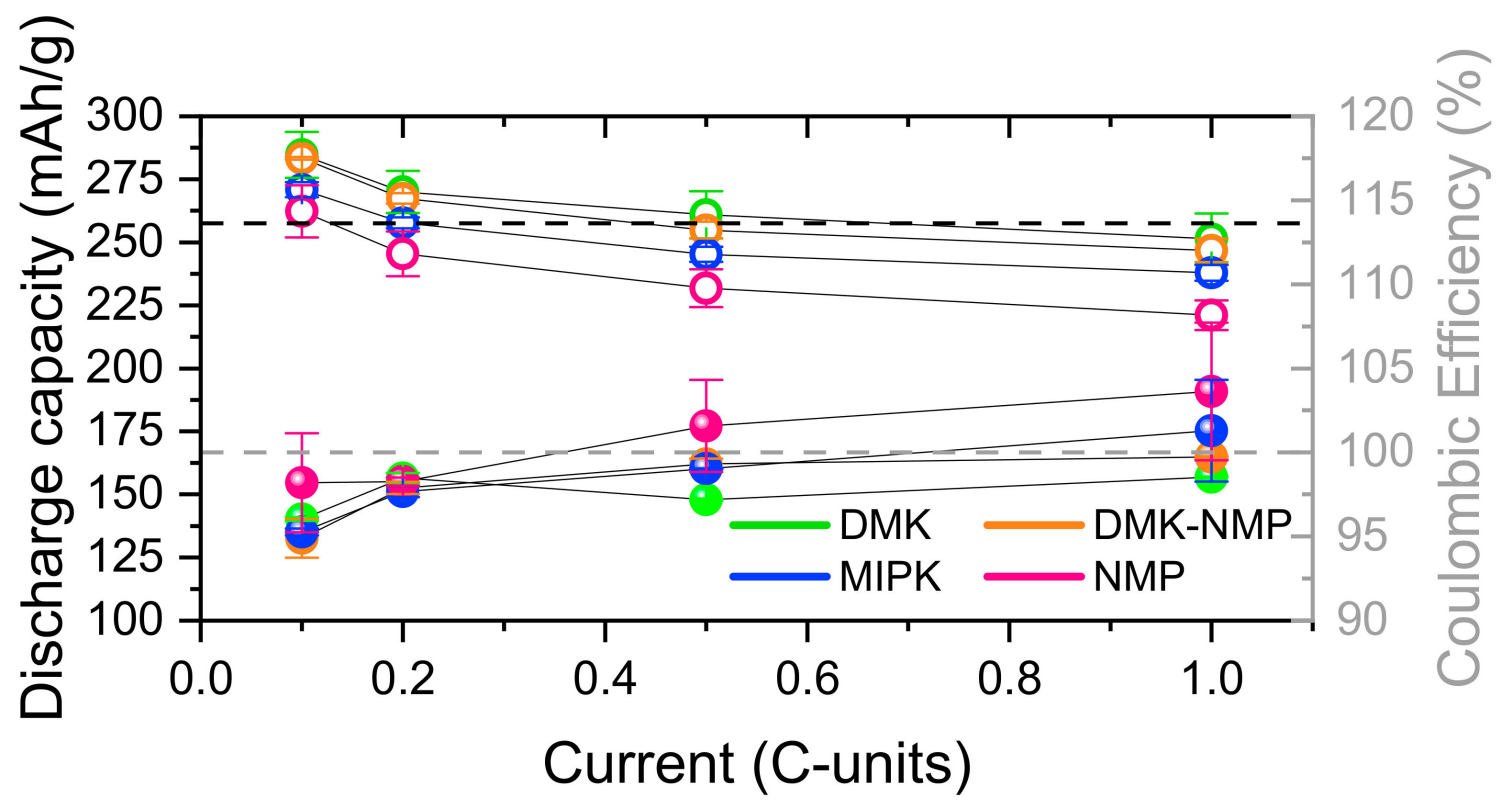
Average of and deviation in specific discharge capacity (open circles) and coulombic efficiency (spheres) vs. current density (C-units) of the four laminates.

The results of the rate capability experiments are summarized in Figure 9 and Figure S10, and Table 4.

The electrochemical performance in the rate capability experiment improved significantly with the cast electrodes compared to the result obtained in powdered form [18]. The powder electrodes consisted of hand-milled mixtures containing 80 wt% active material, 15 wt% Carbon Super C-65, and 5 wt% Ketjen Black. While these results are similar to those recorded in this study at low specific currents (C/10), the capacity dropped below 150 mAh/g at high currents (1C) in the powder electrodes. As shown in Table 4, in our laminates, the capacities at 1C do not fall below 220 mAh/g, with a capacity retention from C/10 to 1C of 87–88% for DMK, MIPK, and DMK-NMP, and of 84% for NMP. This result confirms that processing Schiff base electrodes in laminate form significantly enhances the electrochemical performance in terms of specific rate capability.

As shown in Table 4, the specific capacities obtained from laminates processed with DMK and DMK-NMP are slightly higher than those obtained with MIPK and NMP. This suggests the potential to either substitute NMP with DMK, but its implementation seems challenging due to high-speed evaporation or reduced usage (as in DMK-NMP) in electrode processing. These alternatives are more sustainable, less toxic, and have lower boiling points, leading also to significantly decreased energy costs during the solvent evaporation step. However, for DMK laminates, further research is needed to minimize cracking and improve manageability. The DMK-NMP laminate, which reduces NMP content to 10% (*v*/*v*), emerges as a favorable option due to its high capacity, rate performance, and ease of handling during processing.

#### 3.2.2. Current Collectors

**Figure 10:**
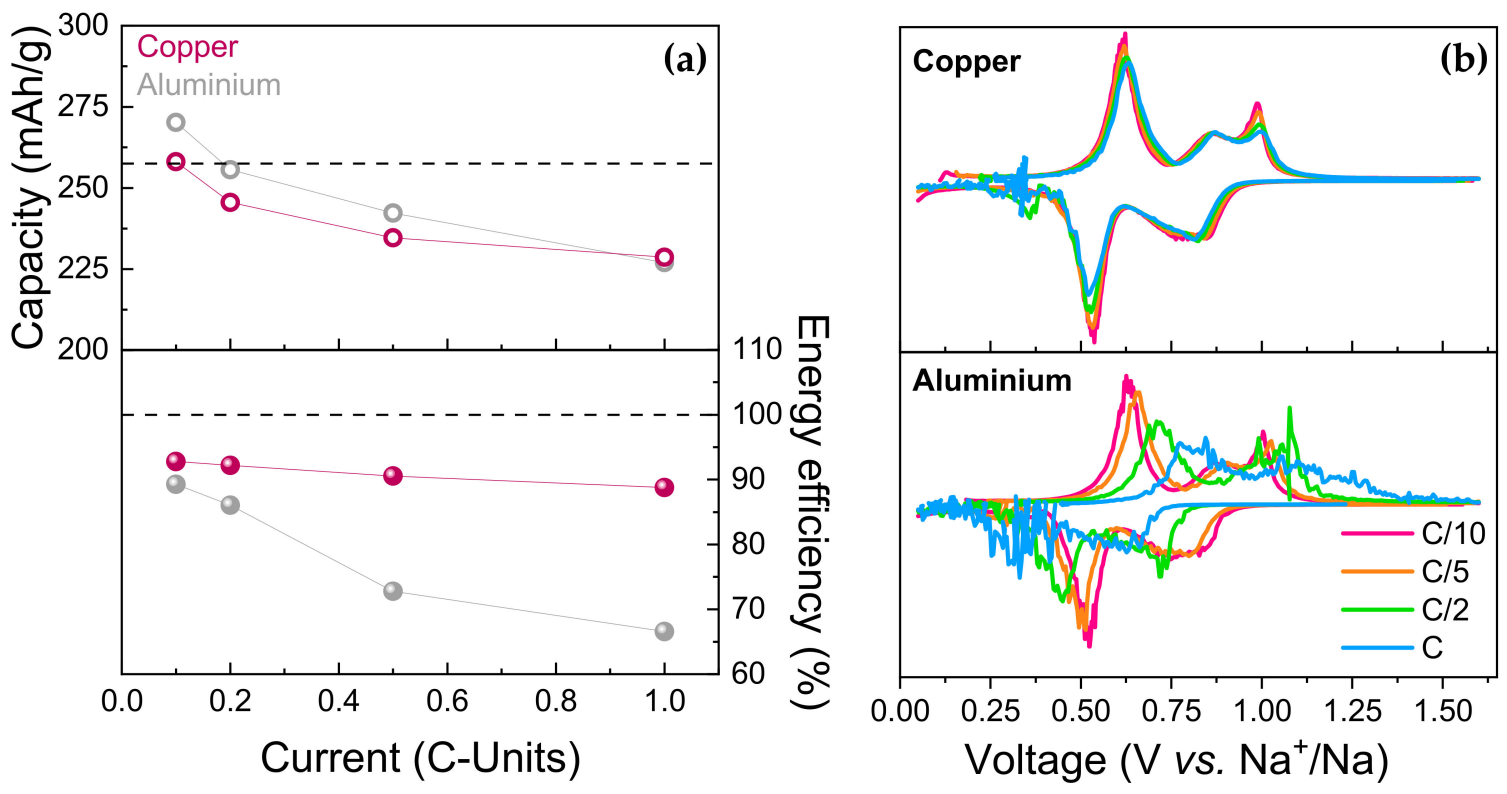
Electrochemical performance of BSNa using copper and aluminum as current collectors. (**a**) Specific capacity (open circles) and energy efficiency (solid circles) vs. current density. Theorical specific capacity (258 mAh/g) and optimum energy efficiency (100%) are marked with dashed lines; (**b**) voltage derivatives of specific capacity vs. potential.

Figure 10 compares the electrochemical performance of BSNa using copper or aluminum as current collectors. As demonstrated in Figure 10a, the capacities for both electrodes are similarly high across varying current intensities. Notably, at low current densities, the capacities are slightly higher with aluminum current collector; however, at high current densities, aluminum shows reduced capacity retention compared to copper. This indicates that while aluminum performs adequately under light current loads with this material, it struggles under heavier loads.

Figure S11 and Figure 10b further elucidate the origin of these differences. When copper is used as the current collector, the redox potentials remain largely consistent regardless of the current intensity. Conversely, with aluminum, increasing current intensity result in higher oxidation potentials and lower reduction potentials. This behavior implies that the redox processes are electronically more hindered with aluminum, affecting the overall battery performance.

Figure 10a presents the energy efficiency of BSNa cells utilizing either copper or aluminum as the current collector. The energy efficiency was calculated as the ratio of energy discharged by the battery to the energy charged into the battery. For these calculations, hypothetical full cells were considered, wherein the positive electrode is a commercial layered oxide NaNi_1-x-y-z_M_x_M′_y_M″_z_O_2_ from Faradion company [34]. This cathode material has a specific capacity of 165 mAh/g and operates at an average voltage of 3.2 V. In this analysis, it is assumed that the average charge and discharge voltages of the cathode are identical, and that changes in the specific energy are only due to differences in the anode voltage polarization and reversible capacity.

Since the energy output of the battery is the product of its capacity and potential, more voltage polarization between charge and discharge implies less energy efficiency. These findings suggest that using aluminum as the current collector for this material leads to lower energy efficiency at high current rates. Figure 10b supports this, showing 5.5 times more loss in energy efficiency from C/10 to C with aluminum (22% loss) compared to copper (4% loss).

Despite these drawbacks, aluminum’s advantages (being lighter, cheaper, and more sustainable than copper), could potentially offset the performance deficits. The lower weight of aluminum reduces the overall weight of the battery, which is beneficial for applications where weight is a critical factor, such as in electric vehicles or portable devices. Additionally, aluminum’s lower cost can lead to significant economic savings, especially in large-scale battery production. Therefore, while aluminum current collectors may exhibit lower performance at high current intensities with BSNa, their cost-effectiveness and lightweight nature could make them a viable alternative to copper, depending on the specific application and performance requirements. Nonetheless, strategies such as carbon coating the Al current collectors [35], which is already established in the cathode current collectors of LIBs, [36] can lead to an improved high rate performance with a small additional weight penalty.

## 4. Conclusions

Bismine sodium salt (BSNa), a Schiff base with two sodium carboxylates, was successfully synthesized through the ion exchange of BSH using NaOH as the sole reagent (acting as the base and cation source), using ethanol as the solvent and avoiding the formation of by-products and the use of toxic reagents. BSNa samples synthesized using this new method yields similar electrochemical results compared to the previous Et_3_N- and NaSCN-based methods. In terms of electrode processing, it has been demonstrated that laminate electrodes perform better than the powdered ones, significantly improving the rate capability with a 84–88% capacity retention upon cycling from C/10 to 1C. Additionally, it was shown that during electrode processing, NMP can be replaced with alternative solvents such as DMK or MIPK or its content can be reduced by 90% in a mixture of DMK-NMP, resulting in a slightly improved electrochemical performance. Finally, the use of aluminum as a current collector for BSNa was evaluated. While aluminum performs similar to copper at a low current density of C/10, it shows a greater decrease in energy efficiency at high current densities compared to copper, 22% vs. 4%, respectively. While the results shown here promote carboxylate Schiff bases as more sustainable anode materials for Na-ion batteries, the challenge to improve its first cycle’s irreversible capacity, which is general in anode materials for SIBs, remains to be tackled.

## Figures and Tables

**Table 1:** Average of the specific capacities (mAh/g) with their deviation for cycles 1 and 2 for BSNa-1, BSNa-2, and BSNa-3.

	Cycle 1		Cycle 2	
	Red	Ox	Red	Ox
BSNa-1	645(17)	237 *	263(3)	236(2)
BSNa-2	708(30)	265(15)	290(14)	263(15)
BSNa-3	755(62)	278(10)	300(11)	276(10)

* Capacity of cell B. Cell A had problems in the oxidation (Figure S4).

**Table 2:** Average of the specific capacities (mAh/g) with their deviation for each rate for BSNa-1, BSNa-2, and BSNa-3.

	C/10		C/5		C/2		C	
	Red	Ox	Red	Ox	Red	Ox	Red	Ox
BSNa-1	247(0)	233(3)	233(1)	227(3)	225(4)	223(4)	211(5)	217 *
BSNa-2	271(13)	260(16)	259(13)	257(19)	250(15)	247(15)	245(16)	245(16)
BSNa-3	285(9)	273(9)	270(9)	266(9)	261(9)	254(9)	251(10)	248(10)

* Capacity of cell B. Cell A had problems in the oxidations.

**Table 3:** Average of the specific capacities (mAh/g) with their deviation for cycle 1 and 2 for the four BSNa-based laminates.

	Cycle 1		Cycle 2	
	Red	Ox	Red	Ox
DMK	755(62)	278(10)	300 (11)	276(10)
MIPK	660(35)	261(3)	280(5)	261(3)
DMK-NMP	857(18)	273(2)	300(1)	272(2)
NMP	804(1)	261(4)	284(5)	259(5)

**Table 4:** Average of the specific capacities (mAh/g) with their deviation for each rate for the four laminates.

	C/10		C/5		C/2		C	
	Red	Ox	Red	Ox	Red	Ox	Red	Ox
DMK	285(9)	273(9)	270(9)	266(9)	261(9)	254(9)	251(10)	248(10)
MIPK	271(3)	258(3)	258(2)	252(3)	245(3)	243(3)	238(3)	241(10)
DMK-NMP	283(1)	269(3)	267(2)	262(3)	255(3)	253(4)	247(5)	246(5)
NMP	262(10)	257(2)	245(9)	241(8)	232(8)	235(1)	220(6)	229(3)

## Data Availability

The raw data supporting the conclusions of this article will be made available by the authors on request.

## Supplementary Materials

The following supporting information can be downloaded at https://www.mdpi.com/1996-1944/17/19/4918/s1: Figure S1: TGA of commercial NaSCN; Figure S2: SEM images of BSNa-1; Figure S3: SEM-EDS mapping of BSNa prepared using the NaSCN method after six washes (a), after thirteen washes (b), and using the NaOH method (c); Figure S4: (a) Voltage vs. specific capacities and (b) voltage derivatives of the specific capacity vs. voltage for cycle (C/10) of BSNa prepared using the NaSCN method after six washes (1), after thirteen washes (2), and using the NaOH method (3). A and B are twin cells; Figure S5: Photograph of laminates prepared with (a) DMK, (b) MIPK, (c) DMK-NMP, and (d) NMP. For scale, the width of the copper foil is 8–10 cm; Figure S6: (a) Infrared spectra and (b) powder X-ray diffraction patterns of the composite electrode powders prepared with the different solvents and BSNa-3 (NaOH method), as well as of BSNa-3 powder; Figure S7: EDX mapping in STEM mode of MIPK laminate; Figure S8: (a) Voltage vs. specific capacities and (b) voltage derivatives of the specific capacity vs. voltage for the first reduction (C/10) of the four laminates; Figure S9: (a) Voltage vs. specific capacities and (b) voltage derivatives of the specific capacity vs. voltage for the first reduction (C/10) of the four laminates; Figure S10: Specific capacities (in mAh/g and normalized, open circles) and coulombic efficiency (spheres) vs. current (C-units) for each cell with the four laminates; Figure S11: Selected charge/discharge curves registered during the rate capability test of BSNa with indicated current collectors.
