# Supplementary material for: Towards More Sustainable Schiff Base Carboxylate Anodes for Sodium-Ion Batteries
Source: Materials (Basel). 2024 Oct 8;17(19):4918. doi: 10.3390/ma17194918 (PMC11605222; doi:10.3390/ma17194918)
Supplement: Supplementary file 1 [file materials-17-04918-s001.zip › materials-3194227-supplementary.pdf]

Supplementary Materials

Towards More Sustainable Schiff Base Carboxylate Anodes for Sodium-Ion Batteries

Irene Gómez-Berenguer [1,2], Bernardo Herradón [2], José Manuel Amarilla [3] and Elizabeth Castillo-Martínez [1,*]

[1] Departamento de Química Inorgánica, Universidad Complutense de Madrid, 28040 Madrid, Spain
[2] Instituto de Química Orgánica General, Consejo Superior de Investigaciones Científicas, 28006 Madrid, Spain
[3] Instituto de Ciencia de Materiales de Madrid, Consejo Superior de Investigaciones Científicas, 28049 Madrid, Spain
* Correspondence: ecastill@ucm.es

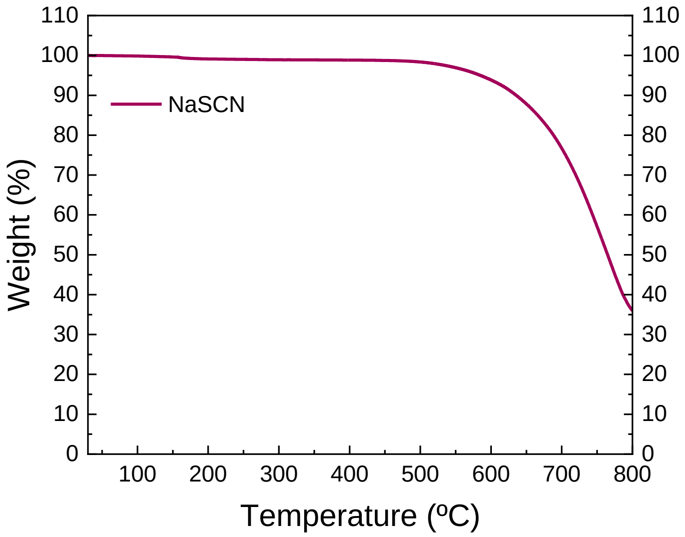

Figure S1. TGA of commercial NaSCN.

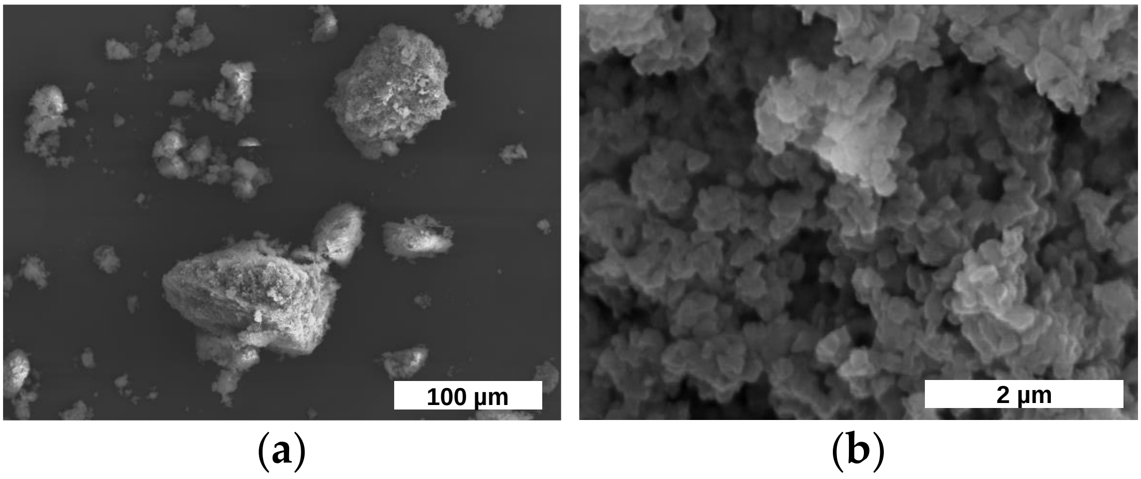

Figure S2. SEM images of BSNa-1.

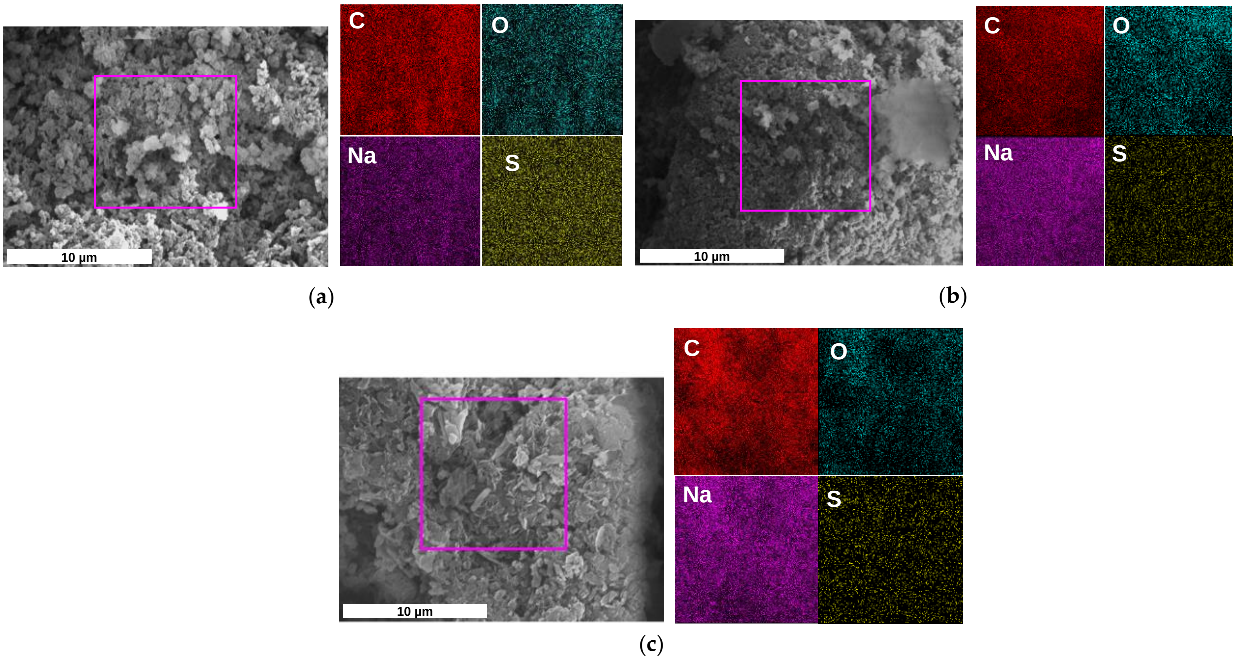

Figure S3. SEM-EDS mapping of BSNa prepared by the NaSCN method after six washes (**a**), after thirteen washes (**b**), and by the NaOH method (**c**).

SEM-EDS mapping cannot accurately determine the presence of sulphur in very small quantities. The mapping indicates similar sulphur levels for the BSNa-3 obtained via the NaOH method and the BSNa-2 from the NaSCN method after thirteen washes. Since the BSNa-3 from the NaOH method contains no sulphur, this signal is attributed to background noise.

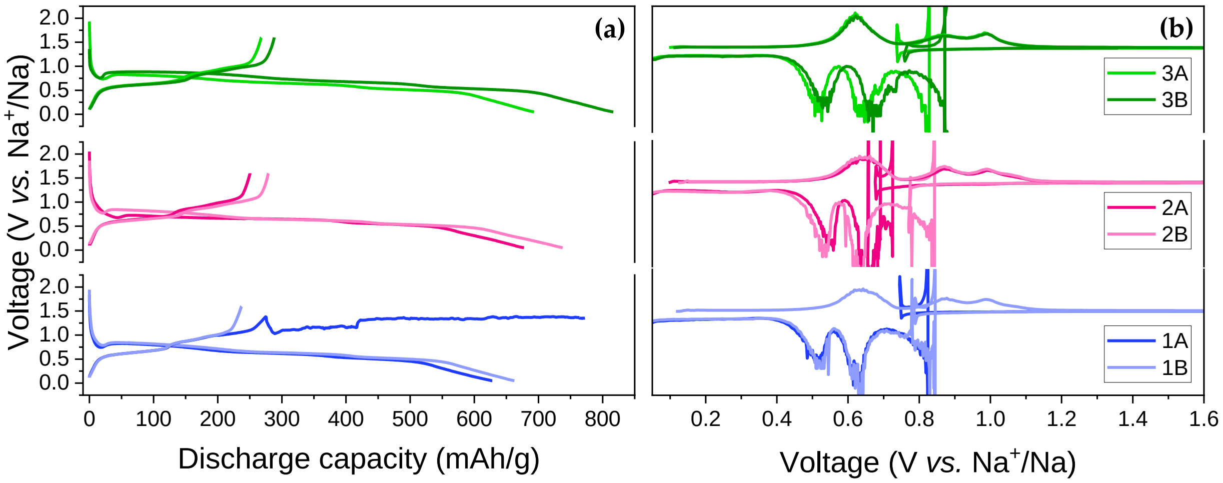

Figure S4. (**a**) Voltage *vs.* specific capacities and (**b**) voltage derivatives of the specific capacity *vs.* voltage for cycle (C/10) of BSNa prepared by the NaSCN method after six washes (1), after thirteen washes (2) and by the NaOH method (3). A and B are twin cells.

In Figure S4b, only the first oxidation of cell B for BSNa-1 has been included due to problems encountered during this step, as observed in Figure S4a. This cell experienced similar issues during multiple oxidations throughout the rate capability experiment.

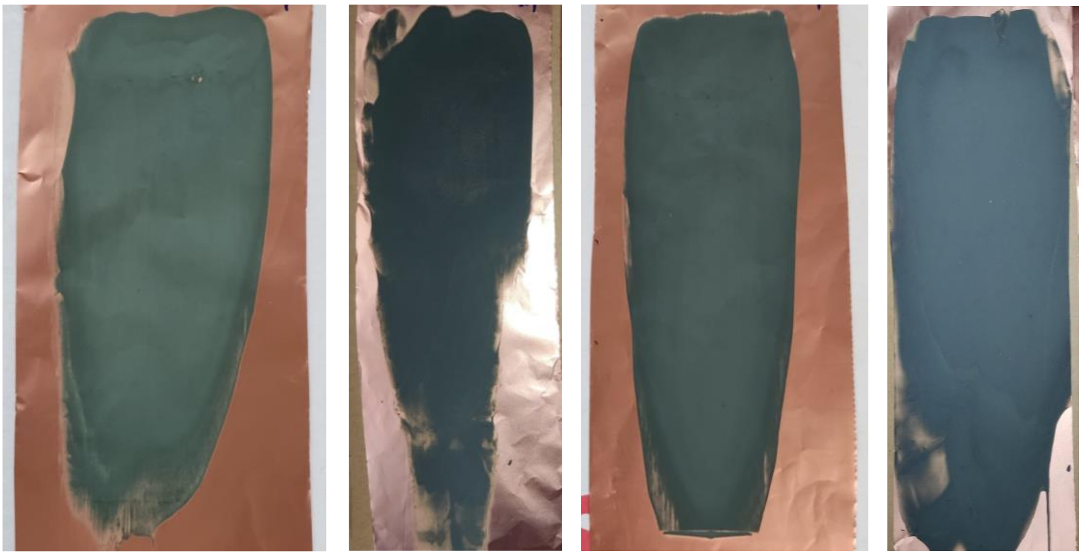

(a) **(b)** **(c)** **(d)**

Figure S5. Photograph of laminates prepared with (**a**) DMK, (**b**) MIPK, (**c**) DMK-NMP and (**d**) NMP. For scale the width of the copper foil is 8-10 cm.

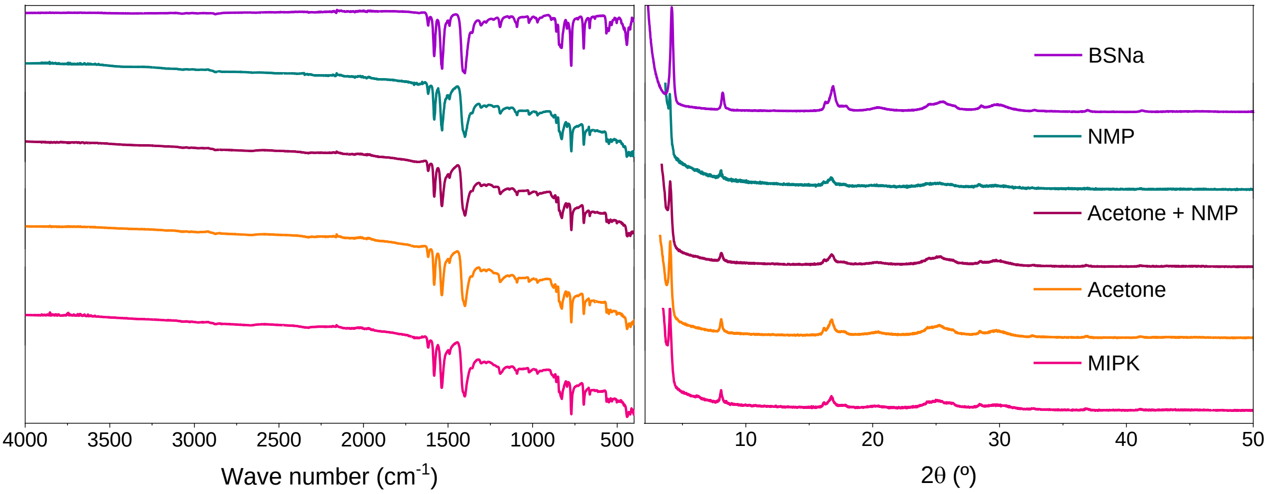

(a) **(b)**

Figure S6. (**a**) Infrared spectra and (**b**) powder X-ray diffraction patterns of the composite electrode powders prepared with the different solvents and BSNa-3 (NaOH method), as well as of BSNa-3 powder.

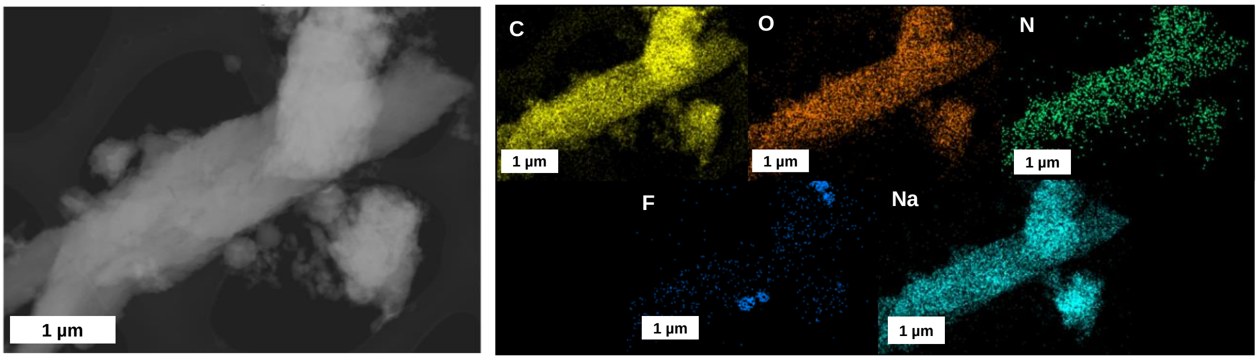

Figure S7. EDX mapping in STEM mode of MIPK laminate.

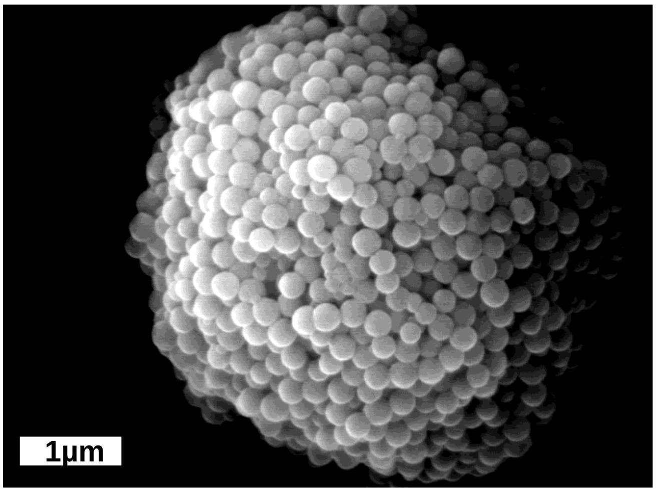

Figure S8. SEM image of commercial PVDF-HFP powder.

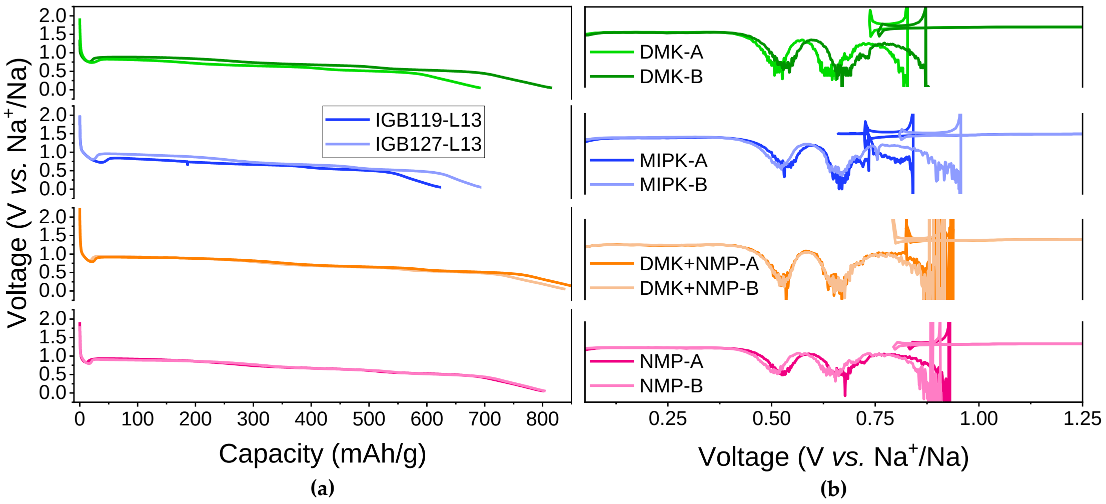

Figure S9. (**a**) Voltage *vs.* specific capacities and (**b**) voltage derivatives of the specific capacity *vs.* voltage for first reduction (C/10) of the four laminates.

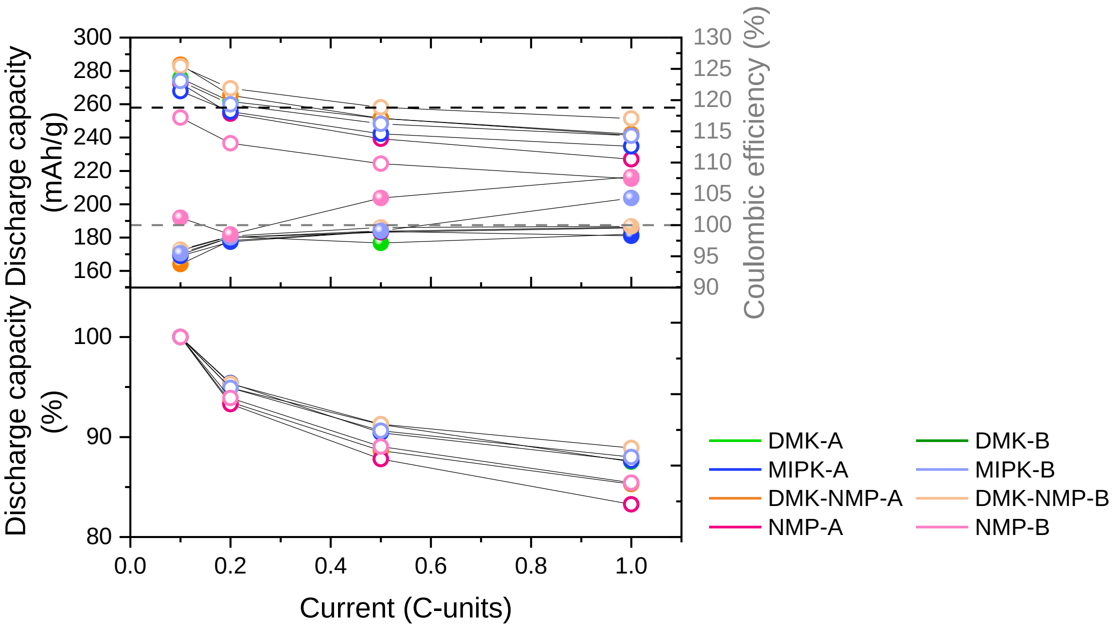

Figure S10. Specific capacities (in mAh/g and normalized, open circles) and coulombic efficiency (spheres) *vs.* current (C-units) for each cell with the four laminates.

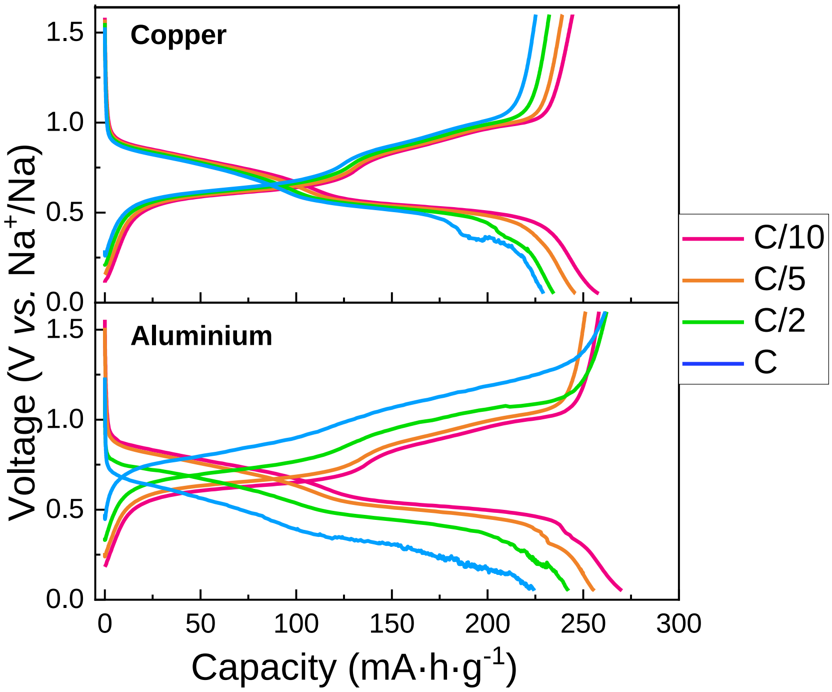

Figure S11. Selected charge/discharge curves registered during the rate capability test of BSNa with indicated current collectors.
